# Super‐recognizers: From the lab to the world and back again

**DOI:** 10.1111/bjop.12368

**Published:** 2019-03-20

**Authors:** Meike Ramon, Anna K. Bobak, David White

**Affiliations:** ^1^ Applied Face Cognition Lab University of Fribourg Switzerland; ^2^ Psychology Faculty of Natural Sciences University of Stirling UK; ^3^ UNSW Sydney New South Wales Australia

**Keywords:** face identification, face matching, face processing, face recognition, super‐recognizers

## Abstract

The recent discovery of individuals with superior face processing ability has sparked considerable interest amongst cognitive scientists and practitioners alike. These ‘Super‐recognizers’ (SRs) offer clues to the underlying processes responsible for high levels of face processing ability. It has been claimed that they can help make societies safer and fairer by improving accuracy of facial identity processing in real‐world tasks, for example when identifying suspects from Closed Circuit Television or performing security‐critical identity verification tasks. Here, we argue that the current understanding of superior face processing does not justify widespread interest in SR deployment: There are relatively few studies of SRs and no evidence that high accuracy on laboratory‐based tests translates directly to operational deployment. Using simulated data, we show that modest accuracy benefits can be expected from deploying SRs on the basis of ideally calibrated laboratory tests. Attaining more substantial benefits will require greater levels of communication and collaboration between psychologists and practitioners. We propose that translational and reverse‐translational approaches to knowledge development are critical to advance current understanding and to enable optimal deployment of SRs in society. Finally, we outline knowledge gaps that this approach can help address.

## Background

Super‐recognizers (SRs) are individuals who are extremely proficient at processing facial identity. In the past decade, it has become clear that people vary in their proficiency on laboratory‐based tasks of facial identity processing (see, e.g., Lander, Bruce, & Bindemann, 2018 for a review). These tests, which typically require participants to discriminate between or recognize previously unfamiliar faces, have demonstrated that face processing ability is characterized by large individual differences with some individuals attaining high performance (e.g., Bobak, Pampoulov, & Bate, 2016; Bowles *et al*., 2009). Moreover, such inter‐ individual differences have been linked to stable genetic factors (Shakeshaft & Plomin, 2015; Wilmer *et al*., 2010).

**Figure 1:**
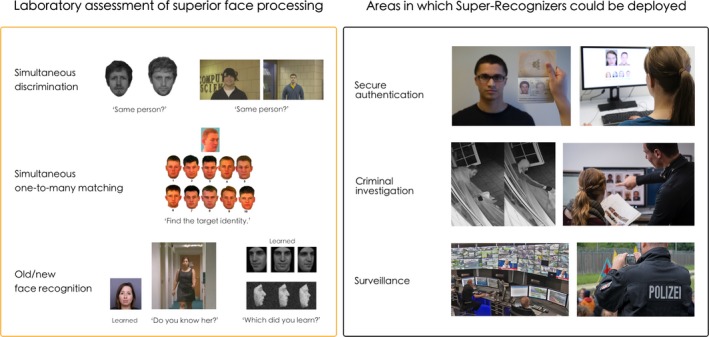
Super‐Recognizer identification in the lab, and potential for deployment in the real world. In laboratory settings (left box), superior face processing abilities are commonly assessed with experimental paradigms involving (top to bottom) simultaneous discrimination of pairs of stimuli (Robertson *et al*., 2016; Phillips *et al*., 2018), simultaneous one‐to‐many matching (Bruce *et al*., 1999), and memory paradigms designed to assess learning of facial identity using videos (left: Bobak et al., 2016, pics.stir.ac.uk) and static images (right: Russell *et al*., 2009). In the real‐world, SRs are selected using lab‐based tests and “on the job performance” (e.g. Davis *et al*., 2016), and have supported criminal investigations (Ramon, 2018a). They could be deployed in a diverse range of operational law enforcement and security settings (right box), including (top to bottom) e.g. passport control, investigative purposes (left image: West Midlands Police, https://www.flickr.com/photos/westmidlandspolice/39164763734/; right: Landespolizei Schleswig‐Holstein Filmgruppe), or crowd surveillance (left: Community Safety Glasgow; right: Landespolizei Schleswig‐Holstein Filmgruppe). [Colour figure can be viewed at wileyonlinelibrary.com]

These discoveries followed decades of empirical work, showing that people in general are poor at processing facial identity of unfamiliar, compared to familiar individuals (e.g., Hancock, Bruce, & Burton, 2001). More recently, studies with professionals trained to perform security‐critical identity verification tasks have shown that they perform no better than students on tasks that are representative of their daily work (Wirth & Carbon, 2017; White, Kemp, Jenkins, Matheson, & Burton, 2014; cf., Figure 1). SRs have been viewed as a solution to this problem, and there is increasing interest in deploying SRs in real‐world settings that stand to benefit from their superior ability, such as policing, national security, and surveillance. For instance, individuals selected based on their face processing abilities have been deployed within the London Metropolitan Police (Davis, Forrest, Treml, & Jansari, 2018; Davis, Lander, Evans, & Jansari, 2016; Robertson, Noyes, Dowsett, Jenkins, & Burton, 2016), as well as the Police in Cologne, Germany.^1^ They have been reported to have assisted investigations of several high‐profile cases, for example, Alice Gross's murder in the United Kingdom,^2^ the recent Novichok poisonings in Salisbury (UK),^3^ and the mass assaults on women in Cologne (Germany) on New Year's Eve 2015.^4^

In concert with the widespread media coverage of SRs in such operational deployments, other initiatives have emerged. The resulting rapid translation of limited scientific evidence into applied practice in this area has sometimes led to an overstatement of the benefits of deploying SRs and unsubstantiated claims. For example, one professional agency recently claimed that ‘Super recognisers can remember 80% of faces they have seen. The average person can only remember about 20% of faces they have seen’^5^ and assure their staff's high ability through ‘vigorous and continued training’.^6^ Another professional association^7^ offers membership accreditation to practice as a SR. Such claims and offers are not corroborated by the limited number of studies of SRs available to date. These have thus far documented a 5–17% point advantage depending on the empirical test used (Davis *et al*., 2016; Robertson, Jenkins, & Burton, 2017). Additionally, several studies report that professionals, whose jobs require frequent image matching, are no better than inexperienced student control participants (Bruce, Bindemann, & Lander, 2018; see also Papesh, 2018; White *et al*., 2014). Finally, it is unclear what an accreditation to practice as an SR entails and in what capacity the associates are encouraged to operate.

Here, we argue that the current level of scientific understanding of superior face processing abilities does not yet warrant broad placement of SRs in diverse operational settings. We briefly outline the present state of scientific knowledge, before highlighting key shortcomings that limit our understanding of the potential benefit of SR deployment. These shortcomings can be attributed to the limited number of available studies examining exclusively the SR population (Table 1) and, hence, our insufficient understanding of the functional basis of superior face processing skills. Additionally, we currently lack a detailed understanding of the real‐world tasks that SRs are expected to perform and whether laboratory‐based tests capture the real‐world abilities of interest (see Figure 1).

**Table 1:** Abilities assessed in studies of superior face processing skill

	IQ	Unfamiliar identity learning / recognition	Unfamiliar identity matching	Famous face identification	Holistic processing	Object processing	Emotion processing	Other non‐identity related face processing	Other
Russell et al., 2009	X	CFMT+	CFPT	BTWF	CFPT IE	X	X	X	X
Russell et al., 2012	X	CFMT+	CFPT	X	X	X	X	X	X
Bobak et al., 2016a	X	CFMT+, recognition from moving footage	1‐in‐10 test	X	X	X	X	X	X
Bobak et al., 2016b	X	CFMT+	CFPT, MFMT, GFMT	X	X	X	X	X	X
Bobak et al., 2016c	WTAR, WASI	CFMT+	CFPT, SMT‐faces	X	CFPT IE, CFE,SMT‐IE	CCMT, SMT‐ hands and houses	X	X	GBI
Bobak et al., 2016d	X	CFMT+	CFPT	X	X	X	X	X	Self‐report, SIAS, STAI‐T,
Davis et al., 2016	X	CFMT+, Old/New UFMT	1‐in‐10 test, GFMT	FFRT	X	Object Memory Test‐flowers	X	X	X
Robertson et al., 2016	X	X	MFMT, GFMT	PLT	X	X	X	X	X
Bobak et al., 2017	X	CFMT+	CFPT	X	X	X	X	X	Eye‐tracking
Bennetts et al., 2017	WASI	CFMT+	CFPT, SMT‐faces	X	CFPT‐IE SMT‐IE	CCMT, SMT‐hands and houses	Ekman 60, RMITE	Age (PFPB), Gender (PFPB)	Eye‐tracking BORB
Bate et al., 2018		CFMT+, MMT	PMT, CMT	X	X	X	X	X	X
Davis et al., 2018	X	CFMT+, SFCT		X	X		X	X	IPIP, NASA‐TLI, CBT
Phillips et al., 2018	X	X	Matching of image pairs	X	X	X	X	X	X
Belanova et al., 2018	X	CFMT+, AFRT, IFRT	X	X	X	X	X	X	EEG

Note

AFRT (Adults Face Recognition Test, Belanvova et al., 2018); BORB (Birmingham Object Recognition Battery, Humphreys & Riddoch, 1993); BTWF (Before They Were Famous, Russell et al., 2009); CBT (Change Blindness Test, Smart et al., 2014); CCMT (Cambridge Car Memory Test; Dennett et al., 2011); CFE (Composite Face Effect Robbins & McKone, 2007); CFMT+ (Cambridge Face Memory Test Long Form; Russell et al., 2009); CFPT (Cambridge Face Perception Test; Duchaine et al., 2007); CMT (Crowd Matching Test; Bate et al., 2018); Ekman 60 (Ekman 60 faces test; Young et al., 2002); FFRT (Famous Face Recognition Test; Lander et al., 2001); GFMT (Glasgow Face Matching Test; Burton et al., 2010); Global Bias Index (Navon, 1977); IE (Inversion Effect); IFRT (Infant Face Recognition Test, Belanova et al., 2018) IPIP (International Personality Item Pool Representation of the NEO PI‐R™; Goldberg, 1998); MFMT (Models Face Matching Test; Dowsett & Burton, 2015); MMT (Models Matching Test, Bate et al., 2018); NASA‐TLI (National Aeronautics and Space Administration Task Load Index Hart & Staveland, 1988); Old/New UFMT (Old/New Unfamiliar Memory Test, Davis et al., 2016); SFCT (Spotting Face in a Crowd Test, Davis et al., 2018); PFPB (Philadelphia Face Perception Battery; Thomas et al., 2008); PLT (Pixelated Lookalike Test; Robertson et al., 2016); PMT (Pairs Matching Test; Bate et al., 2018); RMITE (Reading the Mind in The Eyes; Baron Cohen et al., 2001); SIAS (Social Interaction Anxiety Scale, Mattick & Clarke, 1998); SMT (Sequential Matching Task); STAI‐T (State Trait Anxiety Inventory‐ Trait; Spielberger et al., 1983); WASI (Wechsler abbreviated Scale of Intelligence; Wechsler 1999); WTAR (Wechsler Test of Adult Reading; Holdnack, 2001).

We propose that solving these problems requires researchers and practitioners to approach this growing field of research in a fundamentally different way. The emergence of effective strategies for selecting and deploying individuals with superior face processing abilities requires regular communication between scientists and practitioners. Specifically, we suggest that future research in this area should incorporate a feedback loop encompassing translational and reverse‐translational research – *from the lab to the world and back again* (cf. Ledford, 2008). This is critical for developing robust theory that transfers to an understanding of real‐world tasks and for streamlining recruitment processes and legal guidelines to support the deployment of SRs in society.

### Identifying superior face processing – A solution to real‐world problems?

The concept of SRs was introduced in the seminal work of Russell, Duchaine, and Nakayama (2009) and Russell, Chatterjee, and Nakayama (2012). These researchers found that, relative to a control sample, a group of individuals who self‐identified or were singled out by acquaintances as possessing superior face recognition skills achieved high scores on three tests: the Cambridge Face Memory Test Long Form (CFMT+), Cambridge Face Perception Test (CFPT), and the Before They Were Famous Test (see also Noyes, Phillips, & O'Toole, 2017a for a summary of these tests). Two of these tests (CFMT+, CFPT) were originally developed for the purpose of assessing the face processing performance of people with impaired ability (developmental prosopagnosia; DP). The limited number of studies that have emerged since has primarily aimed to establish whether individuals who excel at these tests also outperform controls at other tasks of face and object processing (for a comprehensive summary of SR studies published to date, see Table 1).

Three important aspects are shared by most laboratory‐based studies on this topic. First, SRs have been identified based on measures originally designed to test face processing at the low‐performing end or normal range of the ability continuum, and it is not clear whether these measures are equally suited to identify high‐performing individuals. Second, while SRs *as a group* tend to outperform groups of non‐SR controls, individual SRs’ performance can be within the average range, and individual SRs present with heterogeneous patterns of performance across different face processing tests (e.g., Bate *et al*., 2018; Bobak, Hancock, & Bate, 2016; Phillips *et al*., 2018; Ramon & Bobak, 2017). This mirrors findings from individuals with DP who lie at the opposite end of the ability spectrum and present with profound deficits in face processing. As a result, this clinical condition^8^ continues to lack a consensus on appropriate diagnostic criteria (see Geskin & Behrmann, 2017). Third, the tests that are used to identify SRs are not representative of the diverse operational tasks that they could encounter if professionally deployed. For example, face images used in standardized tests are classically captured in controlled environmental conditions (e.g., optimal and consistent camera settings) and involve experimental manipulations that are unlike naturally occurring variations (e.g., noise masking, and hair and contour removal). As a result, these tasks may not incorporate those challenges in identity processing that occur in real‐life environments (see Figure 1; cf. Bate *et al*., 2018; Jenkins, White, Van Montfort, & Burton, 2011).

Although previous studies have provided valuable empirical insights, both the cognitive and perceptual basis of superior face processing, as well as the potential translation of laboratory‐based to real‐life performance, remain uncertain. As we outline in the following sections, the development of scientific understanding and solutions is hindered by the current lack of appropriate diagnostic criteria for SR identification and evidence‐based guidelines for effective SR deployment. We argue that a main factor contributing to this status quo is that no studies to date offer a task analysis of the role(s) that SRs play in various organizations. Consequently, the tests used to identify and recruit SRs are not optimized for the specific requirements of varied applied purposes.

We expand on previous findings and recommendations (see Noyes *et al*., 2017b for a recent review), by proposing a framework to assess individual performance using measures that translate *directly* to the ‘process(es) of interest’, that is, those required in real‐life settings. The diverse real‐world tasks SRs are (potentially) expected to perform (see Figure 1) underscore the need to develop selection measures that capture abilities pertinent to these tasks specifically. We contend that greater communication between scientists and practitioners is required to meet the increasing demands for SR deployment in applied settings and to ensure that scientific understanding in this area keeps pace with developments occurring outside the laboratory.

### Quantifying the potential benefits of SRs in applied settings

**Figure 2:**
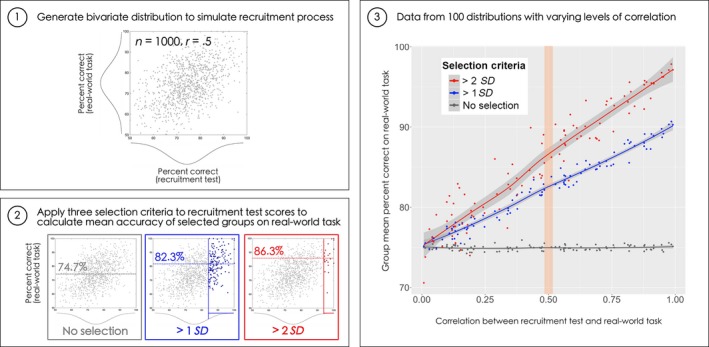
Monte Carlo simulation to estimate the benefit of recruiting SRs. (1) We simulated 100 normal bivariate distributions representing the correlation between a recruitment test and a real‐world task for 1,000 ‘candidates’. The level of correlation between accuracy on the recruitment test and the real‐world task was set randomly for each simulation (.5 in this example). (2) For each of these 100 simulations, three criteria were applied to recruitment test scores in order to select face processing specialists (no selection, greater than one standard deviation above the mean, greater than two standard deviations above the mean). We then calculated the mean accuracy of these groups on the real‐world task. (3) Simulation data showing the mean real‐world accuracy of selected groups for all 100 simulations, as a function of the level of correlation between recruitment test and real‐world task. Estimated benefits of selection are signified by the difference between regression lines for selected groups (blue, red) and the non‐selected group (grey). The orange shaded area represents the ‘best‐case’ correlation between laboratory‐based tests and real‐world tasks based on existing estimates (*r* = .5, see text for details). At this level of correlation, benefits of selection are approximately 8% for >1*SD* and 12% for >2*SD* selection criteria. [Colour figure can be viewed at wileyonlinelibrary.com]

The goal of selecting and deploying SRs in applied settings is to improve the reliability of human performance in real‐life tasks involving processing of facial identity. The hope is that such improvements would make societies safer and fairer by, for example, preventing terrorist events, identity fraud, and wrongful convictions (Figure 1). However, the ultimate success of any recruitment measure depends on its correlation with performance in real‐world tasks. We conducted a Monte Carlo simulation to characterize the relationship between such a correlation and the accuracy gains that can be expected in a hypothetical real‐world task. This simulation is illustrated in Figure 2 and provides an exemplary guide to the magnitude of the real‐world performance gain, which can be expected for any given level of correlation with an ideally calibrated recruitment measure.

This simulation entailed generating normal bivariate distributions with two dimensions arbitrarily labelled as percentage correct on the ‘recruitment test’ and the ‘real‐world task’, respectively. For simplicity, each variable ranged on a scale from 50% to 100% representing the full range of performance expected on a two‐alternative forced‐choice task (chance‐level to perfect accuracy). Operating in simulated conditions, we were able to optimally calibrate the tests to the scale: Means were centred on the midpoint (75%), and distribution parameters were set to span the full range of accuracy – a situation that is unlikely to exist in reality. Using this approach, we simulated 100 recruitment processes each involving 1000 ‘candidates’, in which the correlation between recruitment test and real‐world accuracy varied randomly. To reiterate, our hypothesized recruitment process was modelled as a virtual ‘best‐case’ scenario – with perfectly calibrated measures, and a very large sample to select from.

This approach enabled us to plot the expected gains in performance for each level of correlation, as shown in Figure 2. We computed average real‐world performance of groups containing individuals that scored either >1*SD* or >2*SD* on the recruitment test, with the difference in performance between selected (red, blue lines) and unselected groups (grey line) showing the estimated benefit of the selection criteria. These selection criteria were used to reflect the strict criteria prescribed in the scientific literature (2*SD*) and the fact that many organizations may opt for a more lenient criterion so that they could select larger groups of individuals using other selection measures (e.g., 1*SD*).^9^

We believe the data shown in Figure 2 are informative for decision makers because they provide a guide to the real‐world benefit that can be expected when the level of correlation between a selection measure and performance on a real‐world task is *known*. While the correlation between laboratory‐based and real‐world measures is often difficult to estimate, it is important to quantify where possible. Balsdon, Summersby, Kemp, and White (2018) measured the correlation between the short version of the Cambridge Face Memory Test (CFMT short; 72 items; Duchaine & Nakayama, 2006), the Glasgow Face Matching Test (GFMT), and a task designed to simulate passport issuance officers’ actual task (i.e., reviewing passport image arrays to decide whether any of the images matched the passport applicant). The CFMT short and the GFMT showed correlations with this real‐world task of *r *=* *.41 and *r *=* *.46, respectively.^10^ Note that other studies have reported standardized tests such as the CFMT+ and GFMT as having substantially less predictive value for more complex real‐world tasks, such as spotting a person in a crowd, a task mimicking CCTV surveillance (*r *=* *.18; Davis *et al*., 2018), or perpetrator identification in lineups (Ramon, 2018a). Therefore, considering the available data and diverse range of operational scenarios of SR deployment, a correlation in the range of .4 to .5 would represent an upper estimate.

What does this mean for the selection of specialist teams based on individual face processing ability? As shown in Figure 2, for a laboratory‐to‐world correlation of .5, selecting individuals scoring more than 2*SD* above the mean on a laboratory‐based recruitment measure would result in a real‐world gain of approximately 12%. In practice, however, it is likely that selection will be made from small sets of potential recruits and so it is perhaps more realistic in these cases that less stringent criteria would be used to recruit high performers. For example, if a recruitment process for passport officers involved testing 100 applicants, a 2*SD* selection criterion would produce an average of just two to three successful applicants to choose from. Relaxing selection criteria to 1*SD* above the mean, as potentially necessary in practice, leads to an 8% improvement.^11^ Albeit representing a 32% reduction in *errors* (i.e., reduced from 25% to 17%), selection *alone* clearly cannot solve the problem of high error rates, but can support the development of strategies aiming to improve facial identity processing in applied settings.

Additional gains, however, may be achieved through combination with additional solutions. For instance, in the context of face matching, comparable gains can be achieved by aggregating multiple individuals’ responses (i.e., a ‘wisdom of crowds’ approach; Corbett & Munneke, 2018; Dowsett & Burton, 2015; Jeckeln, Hahn, Noyes, Cavazos, & O'Toole, 2018; Phillips *et al*., 2018; White, Burton, Kemp, & Jenkins, 2013; White, Dunn, Schmid, & Kemp, 2015; White, Phillips, Hahn, Hill, & O'Toole, 2015), and these gains are additive with respect to gains based on recruitment alone (Balsdon *et al*., 2018). Therefore, the most promising approach appears to involve a combination of effective, evidence‐based solutions to produce accurate identity processing systems, such as pairing of state‐of‐the‐art algorithms and high‐performing humans (Phillips *et al*., 2018; Towler, Kemp, & White, 2017).

Of course, the potential benefits of deploying SRs are ultimately determined by the correlation between the recruitment tests used to select them and the real‐world tasks they will be required to perform. As a result, substantial improvement of this correlation is necessary before selection measures can be used alone to solve the problem of error‐prone face identity processing. Likewise, evaluating whether selection processes improve operational performance requires linking performance on empirically developed measures to performance on real‐world tasks. At present, this feedback loop simply does not exist: Specialists are deployed in real‐world tasks – *sometimes* based on laboratory‐developed selection measures – without any ongoing, systematic testing of their operational efficacy. As we outline in the rest of this article, this is a critical shortfall because it curtails efforts to develop tests that capture proficiencies that are pertinent to real‐world performance.

### A framework for measuring superior face processing

#### Face cognition, subprocesses, and experimental assessment

**Figure 3:**
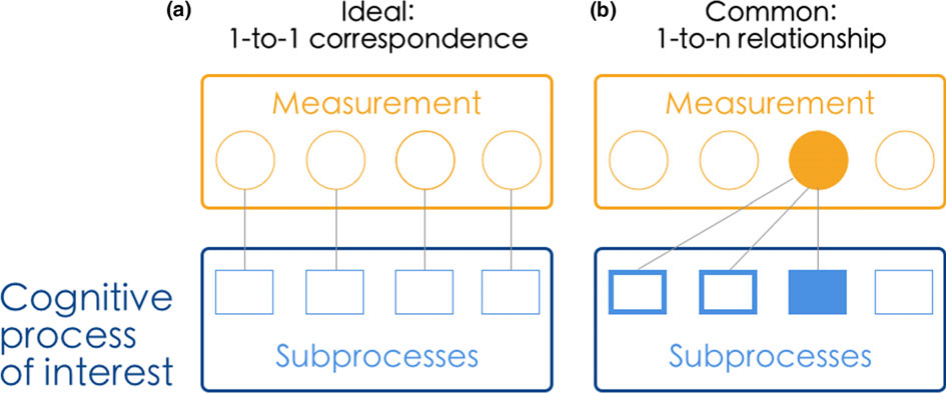
Relationship between cognition and experimental assessment of overt behaviour. (a) A cognitive process of interest, such as face cognition, can involve different subprocesses, which are ideally measured in isolation through dedicated experiments designed to this end. (b) More commonly, experiments designed to measure predominantly one subprocess through observers’ registered responses (filled box) also rely upon additional subprocesses (not filled, thick‐lined boxes), but not others (unconnected box). [Colour figure can be viewed at wileyonlinelibrary.com]

The general process of face cognition, which is presumed to underlie overtly observed behaviour, includes a number of subprocesses, such as face detection, discrimination, recognition, and identification (for a review, see, e.g., Ramon & Gobbini, 2018). Developing effective laboratory‐based measures of superior face processing that are relevant for applied settings necessitates appropriate mapping between the cognitive subprocesses measured in the laboratory and those required in the real world. As illustrated in Figure 3, this is a challenging goal, because any given real‐world task is likely to rely on different subprocesses.

Generally, researchers design experiments with the aim of investigating one or more subprocesses. However, the simple one‐to‐one correspondence between measures and subprocesses illustrated in Figure 3a rarely exists. On the one hand, various different experiments can measure the same process (cf. Hildebrandt, Sommer, Herzmann, & Wilhelm, 2010; Wilhelm *et al*., 2010) with different levels of efficacy. On the other, as exemplified in Figure 3b, one experiment can tap into multiple, but not necessarily *all* existing subprocesses. In this example, a face recognition experiment involves the ability to *detect the presence* of a face, the ability to *distinguish between* faces, and to *recognize* that this person has been seen before. A face *identification* task would involve all of these subprocesses, as well as the ability to retrieve and provide semantic information ‘*This is Meike*’.^12^ Notably, the more subprocesses involved in an experiment, the more difficult it is to control and determine the contribution of each one. For example, superior performance in a face identification task could be observed because of increased ability in discerning or recognizing faces, or retrieving semantic information associated with the face.

Amongst the limited number of SR studies published to date (see Table 1), the majority have identified SR individuals using laboratory‐developed experiments, which measure one or more aspects of processing facial identity – and tap into these subprocesses to presumably varying degrees. Careful consideration of the relationship between subprocesses and utilized measures is particularly critical when creating tests with the intention of identifying SRs for real‐world deployment. Specialists in operational environments often perform diverse tasks that may include familiar face recognition, discrimination of unfamiliar faces, and challenging visual search tasks (Figure 1). Supporting this, a recent study showed that SRs’, facial examiners’, and non‐expert police employees’ performance on laboratory‐based tests did not predict real‐life skills required to identify criminals in lineups after viewing CCTV footage of actual crimes comitted in Switzerland in 2016 (Ramon, 2018a). Because laboratory‐based tests may not be predictive of ecologically meaningful performance (see also Bate *et al*., 2018), measures developed for SR identification for applied purposes should assess subprocesses that mirror their respective professional demands.

Inconsistent or inappropriate terminology usage and neglecting procedural differences further complicates this issue (Ramon, 2018b). To provide a prominent example, the term ‘holistic processing’ has been widely used in the face processing literature. Commonly, it is regarded as the mechanism enabling integration of facial information into a unified percept (cf., e.g., Rossion, 2009). Different experimental paradigms have been used to probe this single theoretical construct, including the part‐whole advantage (Tanaka & Farah, 1993), the face inversion effect (Yin, 1969), and the composite face effect (Young, Hellawell, & Hay, 1987), which can further be implemented in the context of matching, recognition, or identification tasks (see, e.g., Ramon, Busigny, Gosselin, & Rossion, 2016). If all measures of holistic processing tapped into a single common mechanism independent of procedural differences, one would expect them to correlate with one another, as well as with independent measures of face cognition. However, recent evidence suggests that this is not the case. Rezlescu, Susilo, Wilmer, and Caramazza (2017) found that holistic processing measures accounted for little to no variance in CFMT performance (face inversion effect; *r* ^2^ = .18; part‐whole advantage *r* ^2^ = .06; composite face effect *r* ^2^ = .00), and evidence for correlations between holistic processing measures was also weak.

Similarly, superior face processing ability does not appear to be a unitary phenomenon. This is evidenced by the heterogeneous patterns of performance across tests in studies of SRs described above (see Table 1) and also by studies of individual differences in face processing more broadly. The proportion of shared variance (*r* ^2^) between face processing tasks is typically in the range of .10 to .25 and appears to depend on the type of subprocess involved in performing tasks (e.g., Bate *et al*., 2018; Burton, White, & McNeill, 2010; Fysh, 2018; McCaffery, Robertson, Young, & Burton, 2018; Verhallen *et al*., 2017). When considering other abilities that may predict performance in real‐world tasks such as CCTV review and surveillance, this problem is more acute. For example, the ability to match a person based on body cues does not appear to correlate with performance on face identity processing tasks (Noyes, Hill, & O'Toole, 2018), suggesting that face processing tasks are not sufficient to capture abilities that may be pertinent to operational deployment.

We believe this evidence should compel researchers and practitioners to carefully consider subprocesses involved in a given task, as well as the use of precise and appropriate terminology in the context of measuring face processing ability (Ramon, 2018b; Ramon, Sokhn, & Caldara, 2019). The ability of any laboratory‐based test to capture the skill(s) relevant for real‐world tasks will be determined by the extent to which both rely on similar sets of subprocesses (see Figure 3). Moreover, given the varied applied settings of SR deployment, it is unlikely that any single laboratory‐based test will be sufficient and able to identify SRs. This has immediate implications for assessment for SR recruitment and for establishing in‐depth understanding of their underlying abilities.

#### Bridging the laboratory‐world gap to measure ecologically meaningful face processing superiority

To meet the increasing demand for accurate SR identification for real‐world deployment, it is essential to ensure convergence between hypothesis‐driven research and goal‐driven practice. This entails first and foremost identifying practitioners’ goals, which typically exist independently of the theories and models that drive scientific approaches for improving understanding of face cognition.

Over many decades, researchers studying professional expertise have addressed this problem by applying careful analyses of professional tasks. Task‐analytic approaches in professional settings establish a link between a real‐world goal, task, or system, and the cognitive processes that underpin performance (for reviews, see Schraagen, 2006; Schraagen, Chipman, & Shalin, 2000). These techniques have typically been used by applied researchers aiming to improve the design of selection, training, or organizational processes (Schraagen, 2006), and have proven highly beneficial in the development of selection criteria and performance measures in radiology and general medical practice (e.g., Corry, 2011; Patterson *et al*., 2000; Shyu, Burleson, Tallant, Seidenwurm, & Rybicki, 2014). We believe that such practices can serve a similar purpose in the study of superior face processing, by improving the level of correlation between selection measures and the real‐world task (see Figure 2).

This first step – characterizing the real‐world tasks – has been bypassed in SR research. The recruitment of these specialist groups in applied settings has proceeded on the assumption that the laboratory‐based tests – developed by or with scientists – are sufficient to select people that will perform well in real‐world deployment.^13^ However, operational tasks (see Figure 2) can involve complex and diverse challenges, which – in addition to processing of face‐related visual information – may also entail the use of multiple identity cues that are not confined to the face (c.f., Rice, Phillips, Natu, An, & O'Toole, 2013; Noyes *et al*., 2018). As a result, systematic analysis of real‐world tasks should be the starting point for development of recruitment and selection tests. This requires high conceptual precision and adoption of a consistent terminology used to describe tasks and subprocesses involved in face cognition (Ramon, 2018b; Ramon & Gobbini, 2018). Finally, this process should not be performed in a theoretical void, but rather in the light of current scientific understanding of the face processing system, and under consideration of interindividual differences and within‐subject reliability.

**Figure 4:**
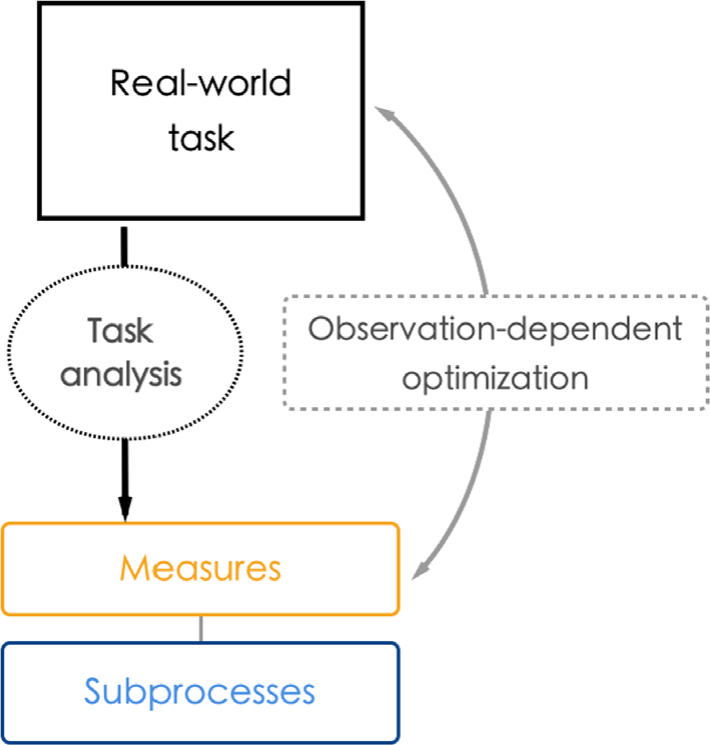
A framework for practice‐oriented development of performance measures. An initial analysis of the real‐world task serves to identify task constraints, practitioners’ goals, and cognitive processes (c.f. Schraagen, 2006). Researchers can then use this information to derive hypotheses about the cognitive subprocesses underlying performance and design experiments to test these hypotheses. This leads to the development of measures, which can be optimized to capture the real‐world task through additional task analyses, and the observed correspondence between accuracy in the measures and performance in on the real‐world task. This process serves to increase the predictive power of tests in terms of predicting performance in real‐world settings. [Colour figure can be viewed at wileyonlinelibrary.com]

Based on these considerations, we suggest an approach to successful development and validation of appropriate assessment measures, as schematized in Figure 4. The first step entails effective task analysis. Having identified the relevant subprocesses, experiments and performance measures can be developed to evaluate exhibited behaviour. Careful, *direct* observation of individual performance (as opposed to, e.g., uncontrolled online testing) is particularly relevant during the initial stages of test development and can provide critical insights regarding the validity of underlying assumptions and limitations of the test design.^14^ Were real‐world tasks and practitioners’ goals translated appropriately into subprocesses and most suitable experiments? Do the tests developed capture distinct subprocesses that underpin real‐world performance? Are they internally consistent? Which factors can account for unexpected observations? Answering these questions in the context of a task‐analytic approach can ensure that experimental tasks are developed appropriately and optimized in alignment with the real‐world tasks.

### From the laboratory to the world and back again

In this article, we have identified three main knowledge gaps in understanding of SRs: (1) the overlap between laboratory‐based tests and real‐world performance; (2) the range of tasks that SRs are expected to perform; and (3) the subprocesses of face cognition underpinning the real‐world tasks and, by extension, novel laboratory assessments of these tasks. We propose that greater synergy between researchers and practitioners is necessary in order to address the shortfall in understanding. In this section, we ask how this should be addressed, describe ongoing efforts to this end. And outline how this can be improved in the future.

**Figure 5:**
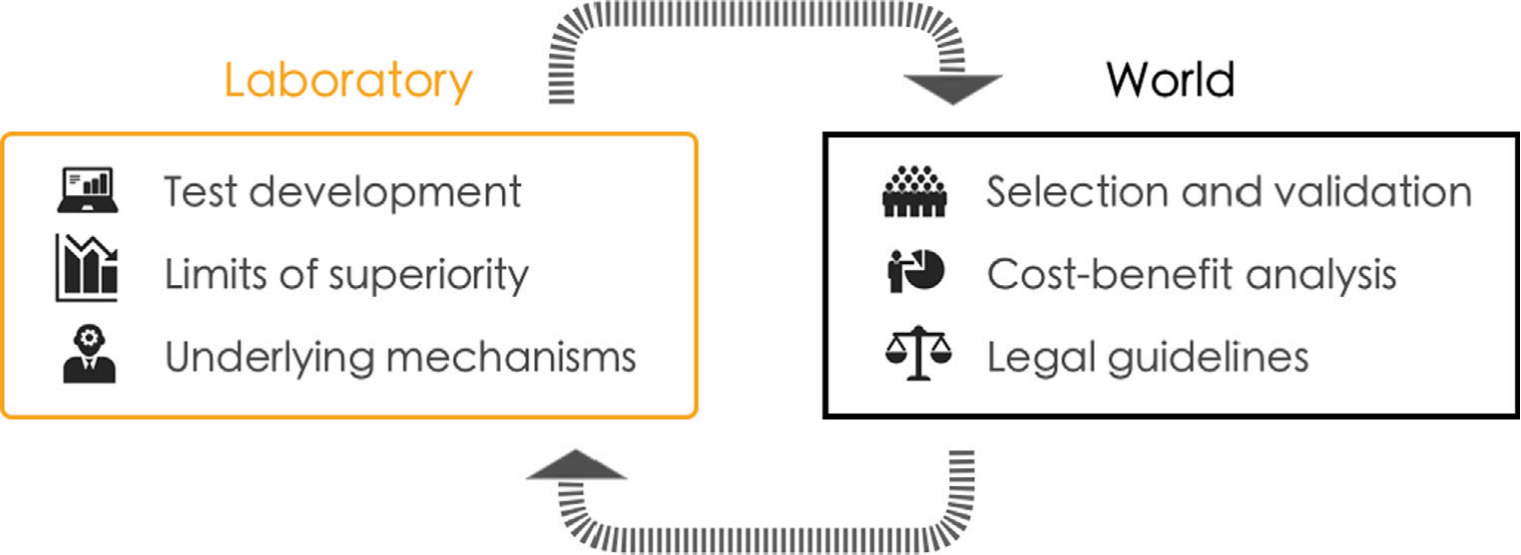
Continued exchange between scientists and real‐world practitioners. This continued development cycle can serve to improve theoretical knowledge of superior face processing, which can in turn help to generate improved processes deployed in professional settings. [Colour figure can be viewed at wileyonlinelibrary.com]

Figure 5 schematizes our proposed knowledge development cycle between the laboratory and the world. In one direction, knowledge emerges from the laboratory: Scientists design studies to understand the underlying mechanisms and the boundary conditions of SRs’ superior performance. This understanding provides the basis for procedures that can be used by practitioners to, for example, select SRs for real‐world deployment, evaluate the potential benefits of using SRs in their organization, and develop guidelines for interpreting evidence provided by SRs in court. Critically, knowledge transfer in the opposite direction – from the world to the laboratory – will advance understanding by attuning scientific procedures to real‐world constraints, for example through real‐world task analyses, and performance data that evaluate the effectiveness of SR deployment. This continuous feedback loop stands to benefit both scientists and practitioners alike, enabling development of better selection measures and improving conceptual and theoretical understanding.

This diagram is useful as a high‐level outline, but what practical measures can be taken to facilitate this knowledge cycle? In recent years, three main mechanisms have emerged. First, scientific working groups devoted to developing best practice guidelines for face identification have been established, and academics have begun to engage with these groups (e.g., NIST Face Identification Subcommittee).^15^ Second, meetings led by academics have been jointly attended by psychologists, computer scientists, forensic scientists, lawyers, police, and employees of various government agencies.^16^, ^17^ Third, collaborative projects between academics and practitioners have been critical in transferring the initial laboratory work to applied settings. These include projects aiming to improve performance in applied settings and to benchmark accuracy of face identification professionals against the members of the public and SRs (e.g., Davis *et al*., 2016, 2018; Phillips *et al*., 2018; Robertson *et al*., 2016; White *et al*., 2014; White, Dunn, *et al*., 2015; White, Phillips *et al*., 2015). Collaborations with international police agencies have also begun to address issues pertaining to SR selection (North Rhine‐Westphalia Police, Germany), prevalence of SRs based on identification with real‐world tasks in large‐scale professional populations (Berlin Police, Germany),^18^ and deployment of SRs and forensic facial examiners in the context of criminal investigation in Switzerland (Ramon, 2018a).^19^

These developments have served to link the work of researchers and practitioners in a meaningful way and have fostered a network that can form the basis for future translational research. While encouraging, it is critical that these initial steps are followed up by a coordinated approach in the years ahead. Currently, there are very few formal collaborations between practitioners and academics, despite intense interest in this area from both groups, which may lead to demand for SRs in applied settings outpacing scientific understanding of their abilities. This demand has clearly grown in recent years and as a consequence professional associations are emerging that offer memberships, accreditations, and professional opportunities.^20^ In order to facilitate the framework we have outlined in Figure 5 and increase their credibility, it is imperative that such organizations allow their means of selection to be scrutinized by the wider scientific community, by making their accreditation criteria transparent and publicly available; any SR‐related claims must be rooted in data from peer‐reviewed empirical investigations. Such transparency would facilitate simultaneous progress of scientific research and practice in applied settings alike.

A coordinated approach is also necessary in order to establish the potential role of SRs in the legal system. Although we are not aware of SRs providing expert identification evidence in court, there are reports that they have provided evidence as regular police witness in the United Kingdom (Edmond & Wortley, 2016; p. 492). Indeed, it has been suggested that SRs may be an improvement on the current face identification experts that are regularly required to provide expert evidence in court (e.g., Edmond & Wortley, 2016). In this context, the question of whether SRs are superior on real‐world tasks is critical to assessing SRs claims of expertise in court. Indeed, a recent study reported that groups of SRs exhibit performance comparable to groups of professional forensic facial examiners in same/different face matching of frontal images (Phillips *et al*., 2018). This result raises the possibility that SRs could provide evidence in legal trials that is of comparable quality to that of officially trained professionals.

Recent work also indicates that combining the expertise of professionally trained practitioners and SRs with naturally occurring superior face processing skills could provide complementary benefits to the accuracy of facial forensic evidence. SRs seem to require significantly less time to achieve performance comparable to that of facial examiners (Phillips *et al*., 2018), possibly because they do not rely on a piecemeal processing strategy. In turn, forensic facial examiners receive substantial training and mentorship in applying a feature‐based approach for facial image comparison (see Facial Identification Scientific Working Group, 2012), and behavioural tests indicate a greater reliance on analytic, ‘piecemeal’ approaches that differ qualitatively from those of novices and SRs (Towler et al., 2017; White, Dunn, *et al*., 2015; White, Phillips *et al*., 2015). In addition to benefits from combining these dissociable sources of expertise, incorporating SRs within the framework of forensic science may also bring increased legitimacy to the use of SRs in legal practice. For instance, the high level of transparency in working groups that develop best practice in training, tools, and procedures used by forensic facial examiners could be a useful model for developing similar guidelines for testing and deploying SRs.^21^

## Conclusions and future directions

In the last decade, research into superior face processing abilities and the deployment of SRs have emerged and progressed independently. Here, we have identified problems with this approach that hinder progress in both areas, and we propose some initial solutions. In this final section, we acknowledge recent work that has begun to address the issues we have discussed and outline key questions and future challenges.

Recently, a clear focus on developing tests that represent some of the diverse operational deployments of SRs has emerged. Deviating from early tests designed to capture broad aspects of face identity processing abilities such as perceptual discrimination (Burton *et al*., 2010) and memory (Russell *et al*., 2009), more recent work has begun to test SRs on tasks that involve matching image‐based facial memories to video footage (e.g., Bobak, Hancock, *et al*., 2016; Davis *et al*., 2018), in‐crowd identity search (e.g., Bate *et al*., 2018), and face matching (Bobak, Dowsett, & Bate, 2016; Phillips *et al*., 2018) in conditions considered more similar to task demands faced in applied settings.

As we have outlined in this article, it is critical to establish the extent to which performance on laboratory‐based tests will likely generalize to real‐world tasks. This aspect has also been proposed as one of seven ‘action points’ for future SR research (Noyes *et al*., 2017b). Amongst the important principles identified to guide future work in this field, the authors suggested that reaching a consensus on a standard approach to measuring and defining superior face processing abilities is imperative.

From an academic perspective, we agree with this proposal, as it is theoretically possible to optimize measurements in such a way that they ensure identifying the most apt individuals across different subprocesses of face cognition. However, as we have argued here, even if such a scientific consensus on ideal experimental assessment were achieved, this would be unlikely to provide an ‘all‐purpose’ set of measures relevant for long‐term real‐world deployment. The diverse demands of real‐world challenges relating to face processing make it very unlikely that a standard approach to identifying SRs for applied settings could be established. Moreover, any such measures would require regular reviews and updates to continuously match the changing real‐life operational demands.

In our opinion, it is therefore necessary for this emerging research field to approach the complex topic that is SR identification in a systematic and coordinated fashion. On one hand, there is the realistic scenario that independent research groups could each develop their own tests to model a specific operational task. The danger of such an approach would be ‘overfitting’ tests to specific tasks, thereby hindering the goal of defining universal criteria for super face processing abilities. On the other hand, it is clear that the currently available laboratory‐based tests do not adequately capture the diversity of real‐world tasks. We believe that the only solution to this problem is for scientists and practitioners to reach a consensus on the roles that SRs can be useful for and agree on the best set of measures to identify the most promising individuals to fulfil them.

## Footnotes

1
https://www.ksta.de/koeln/koeln-archiv/-ich-schlage-jeden-computer--wie-ein-super-recogniser-der-koelner-polizei-arbeitet-28162628.

2
https://www.newstatesman.com/politics/uk/2016/08/super-recognisers-scotland-yard.

3
https://www.telegraph.co.uk/news/2018/08/28/police-use-super-recognisers-hunt-salisbury-poisoners/.

4
https://www.landtag.nrw.de/portal/WWW/dokumentenarchiv/Dokument/MMD16-14450.pdf.

5
http://superrecognisersinternational.com/agency (Date accessed: 31/11/2018).

6
https://www.linkedin.com/pulse/afr-human-verification-centres-kenneth-long-fsra-qii/?published=t (Date accessed: 31/11/2018).

7
https://www.associationofsuperrecognisers.org/mission (Date accessed: 31/11/2018).

8
https://www.nhs.uk/conditions/face-blindness/.

9 This may be the case, for example, in situations like border control, which requires processing large volumes of identity matching decisions, but where recruitment is based on a variety of selection measures that are not only related to face processing ability (e.g., experience and character).

10 Note that most studies of superior face processing abilities have used the more commonly used CFMT+, which comprises 102 items and therefore yields more reliable individual scores.

11 Binomial effect size display analysis (Rosenthal, 2005) can also be used to estimate the effectiveness of recruitment processes. For example, if one is selecting individuals that perform above average on a recruitment test with a correlation of .5 to the real‐world task, 75% of the recruits will perform above average on the real‐world task.

12 Note that in some contexts, the term ‘face identification’ is used an umbrella term for various tasks that involve processing of facial identity. Throughout this article, we adopt terminology outlined in a recent paper (Ramon & Gobbini, 2018) in order to clearly delineate different subprocesses of face cognition.

13 Indeed, some tests that have been sold to government agencies have not been peer‐reviewed and can therefore not be evaluated (see: https://www.polizei.bayern.de/muenchen/news/presse/aktuell/index.html/281026).

14 For example, laboratory‐based assessment provides an optimal environment for test development by minimizing confounding variables, which are impossible to exclude during online testing (e.g., interference or help from others, variations in stimulus presentation parameters, or technical nuisance factors).

15
https://www.nist.gov/topics/forensic-science/facial-identification-subcommittee.

16
http://forensic.psy.unsw.edu.au/ufig.html.

17
https://www.nist.gov/news-events/events/2018/11/international-face-performance-conference-ifpc-2018.

18
https://www.radioeins.de/programm/sendungen/die_schoene_woche/_/lka-berlin-sucht-den--super-recognizer-.html.

19
https://www.moz.de/nachrichten/brandenburg/artikel-ansicht/dg/0/1/1679849/.

20
https://www.associationofsuperrecognisers.org.

21
https://www.nist.gov/topics/forensic-science/facial-identification-subcommittee.
